# Translating the Dutch Walking Stairs, Walking Ability and Rising and Sitting Questionnaires into German and assessing their concurrent validity with VAS measures of pain and activities in daily living

**DOI:** 10.1186/1471-2474-11-108

**Published:** 2010-06-01

**Authors:** Carolin Heitz, Lucas M Bachmann, Anne Leibfried, Rudolf Kissling, Alfons GH Kessels, Roberto SGM Perez, Johan Marinus, Florian Brunner

**Affiliations:** 1 Department Physiotherapy, Balgrist University Hospital, Zurich, Switzerland; 2 Horten Centre for patient oriented research, University of Zurich, Zurich, Switzerland; 3 Department of Physical Medicine and Rheumatology, Balgrist University Hospital, Switzerland; 4 Clinical Epidemiology and Medical Technology Assessment, University Hospital Maastricht, Maastricht, Netherlands; 5 Department of Anaesthesiology, VU University Medical Center, Amsterdam, Netherlands; 6 TREND (Trauma Related Neuronal Dysfunction) consortium http://www.trendconsortium.nl/home-en; 7 EMGO Institute for Health and Care Research (EMGO), VU University Medical Center, Amsterdam, Netherlands; 8 Department of Neurology, Leiden University Medical Center, Leiden, Netherlands; 9 Department of General Practice, AMC University of Amsterdam, Amsterdam, Netherlands

## Abstract

**Background:**

The Dutch Walking Stairs, Walking Ability and Rising and Sitting Questionnaires are three validated instruments to measure physical activity and limitations in daily living in patients with lower extremity disorders living at home of which no German equivalents are available. Our scope was to translate the Walking Stairs, Walking Ability and Rising and Sitting Questionnaires into German and to verify its concurrent validity in the two domains pain and activities in daily living by comparing them with the corresponding measures on the Visual Analogue Scale.

**Methods:**

We translated the Walking Stairs, Walking Ability and Rising and Sitting Questionnaires according to published guidelines. Demographic data and validity were assessed in 52 consecutive patients with Complex Regional Pain Syndrome 1 of the lower extremity. Information on age, duration of symptoms, type of Complex Regional Pain Syndrome 1 and type of initiating event were obtained. We assessed the concurrent validity in the two domains pain and activities in daily living by comparing them with the corresponding measures on the Visual Analogue Scale.

**Results:**

We found that variability in the German Walking Stairs, Walking Ability and Rising and Sitting Questionnaires was largely explained by measures of pain and activities in daily living on the Visual Analogue Scale.

**Conclusion:**

Our study shows that the domains pain and activities in daily living are properly represented in the German versions of the Walking Stairs, Walking Ability and Raising and Sitting Questionnaires. We would like to propagate their use in clinical practice and research alike.

## Background

Complex Regional Pain Syndrome (CRPS) is a painful condition that often results in substantial disability [1]. Two types of CRPS can be distinguished: type 1, formerly known as reflex sympathetic dystrophy or algodystrophy, which occurs without a definable nerve lesion and type 2, formerly called causalgia, in which a definable nerve lesion is present [2].

In the past the focus of CRPS research was mainly on symptoms and pain. Little attention has been given to the disabilities associated with CRPS. As a consequence, little information is available on the problems CRPS patients encounter in activities in daily living, and specific measurement instruments to address these problems are lacking [3].

We are only aware of one instrument, which allows measuring the functional limitation of CRPS patients. In 2000, Oerlemans et al. developed and validated the Radboud Skills Questionniare (RASQ) to map alterations in the level of disability in patients with CRPS of the upper extremity [4]. Today the RASQ is available in Dutch [4], English (not validated yet) and German language. Various instruments are available to measure activity limitations of the lower extremity [5-10], but we are not aware of a corresponding questionnaire for patients with CRPS 1 of the lower extremity. Most of these existing instruments do not provide a detailed measurement of activity limitation of the lower extremity perceived by the patients. Between 1996 and 2005 a Dutch group of researchers developed and validated three separate tools in Dutch assessing walking ability, including walking stairs, as well as rising and sitting [11-14]. These instruments were applied in various lower extremity disorders such as osteoarthritis, amputation, diabetic foot problems and CRPS 1. These instruments serve as disability measures in a large Dutch CRPS research consortium http://www.trendconsortium.nl. Up to now, these instruments are only available in Dutch and English (not validated). In this paper we describe how we translated the Walking Stairs, Walking Ability and Rising and Sitting Questionnaires into German and how we verified its concurrent validity in the two domains pain and activities in daily living by comparing them with the corresponding measures on the Visual Analogue Scale (VAS).

## Methods

### Recruitment sources and data acquisition

We recruited patients from the outpatient clinic of Balgrist University Hospital, Zurich, Switzerland and through advertisements posted on two self-help homepages for patients afflicted with CRPS (http://www.morbus-sudeck.ch, http://sudeck.foren-city.de). We included all eligible and consenting adult patients suffering from CRPS 1 of the lower extremity with fulfilled International Association for the Study of Pain (IASP) criteria, more than 18 years of age, illness duration of more than three months and the ability to complete the questionnaires. The study protocol was approved by the local Ethics Committee (Spezialisierte Unterkomission für Orthopädie der Kantonalen Ethikkommission, Zurich, Switzerland) and informed consent was obtained from all participants.

### Assessment instrument

The Walking Stairs [14], Walking Ability [13] and Rising and Sitting Questionnaires [11,12] aim at determining perceived activity limitations in patients with lower extremity disorders. They are self administered questionnaires including a total of 79 dichotomous items. The scores of the specific three subdomains can be calculated as well as the total score of all three questionnaires.

### Translation process

**Figure 1:**
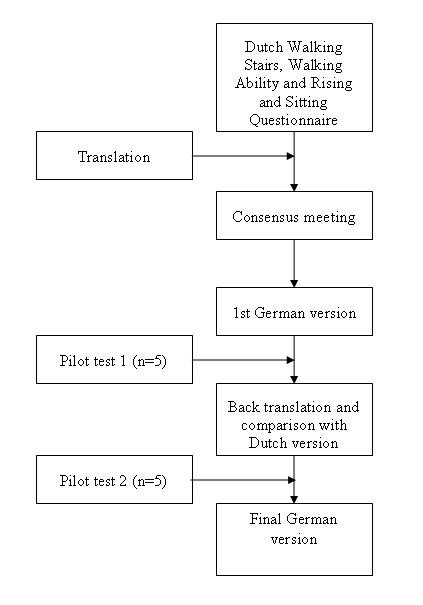
**Flow diagram of the development process of the German Walking Stairs, Walking Ability and Rising & Sitting Questionnaires**.

We followed a sequential forward and backward translation approach (see figure 1) [15]. Two professional translators translated the original Dutch version of the Walking Stairs, Walking Ability and Rising and Sitting Questionnaires into German. In a consensus meeting a rheumatologist, a specialist in physical medicine and rehabilitation, a physical therapist and an epidemiologist assessed the consistency of the translation and judged its face validity. They then agreed on the first German version for these formats. The questionnaires were pilot tested in five CRPS 1 patients to identify difficulties in comprehension and interpretation of the questions. In addition, we tested various possible wordings of items, answer choices and instructions if the translation team considered more than one possible version. A Dutch translator with experience in biomedical sciences but unaware of the original versions performed a backward translation of the German version into the source language (Dutch). A team of experts (a rehabilitation specialist, a rheumatologist, an epidemiologist and a physical therapist) compared the back translation with the Dutch versions to check for conceptual discrepancies. After a second pilot test (n = 5 CRPS 1 patients), the translation team discussed the comments from these patients and decided in consensus on modifications. Finally, the experts approved the final German version of all three questionnaires.

### Validation process

All three questionnaires were offered to CRPS 1 patients meeting the inclusion criteria. Patients received the questionnaires either during a visit in our outpatient clinic or by mail. Participants were asked to complete the questionnaires during the same day and to mail them back to our institution. In order to assess the concurrent validity of the questionnaires, we assessed pain and self perceived restriction in activities in daily (ADL) living on the Visual Analogue Scale (VAS) (0 = no pain/restriction, 10 = worst pain/maximal restriction). We hypothesized, that a higher score on the VAS (pain and ADL) is associated with a more severe functional impairment in CRPS 1 patients.

### Statistical analysis

Values are reported as mean ± SD, medians and interquartile ranges (IQR) or as absolute number and percentage. Linear regression analysis and mean prediction interval were used to assess the relationship between the three questionnaires and the VAS pain respectively VAS of activities in daily living. A probability value of p < 0.05 was considered statistically significant for all tests. We performed all statistical analyses with the SPSS 12 statistical software package (SPSS Inc. Headquarters, 233 S. Wacker Drive, 11th floor Chicago, Illinois 60606).

## Results

### Translation and instrument development

The wording of the questions and response options correspond to the original version. We did not add or remove items nor changed response categories.

### Demographic and clinical characteristics

The demographic and clinical characteristics of the participants are shown in table 1. We enrolled 52 patients suffering from CRPS 1 of the lower extremity (females/males: 46/6). Forty patients (76.9%) suffered from CRPS 1 of the foot and 11 (21.2%) from the knee. Trauma (48.1%) and surgery (46.2%) were the most common initiating events. Median disease duration was 2.2 years (IQR 0.79 to 5.19).

**Table 1:** Demographic and clinical characteristics of study population (N = 52)

Characteristic	Value
Gender	
Male	6 (11.5%)
Female	46 (88.5%)
Mean age (+standard deviation)	50.3 + 14.5 years
Age range	18.2-76.7 years
Affected body part	
Foot	40 (76.9%)
Knee	11 (21.2%)
Other	1 (1.9%)
Median (IQR)* of number of years with CRPS 1	2.2 years (0.79-5.19)
Initiating event	
Trauma	25 (48.1%)
Surgery	24 (46.2%)
Other	3 (5.8%)

* IQR: Interquartile range

### Descriptive statistic of pain and activity limitation on Visual Analogue Scale and the Walking Stairs, Walking Ability and Rising and Sitting Questionnaires

Self perceived pain and restrictions in activities in daily living were 5.7 + 2.1 and 5.6 + 2.2 on the VAS. Average of the total score of the questionnaires was 29.4 + 13.2 (walking stairs 5.6 + 2.5, walking ability 11.9 + 5.6, rising and sitting 11.9 + 6.5). For the detailed analysis see table 2.

**Table 2:** Descriptive statistics of pain and activity limitation on Visual Analogue Scale, and the Walking Stairs, Walking Ability and Rising and Sitting Questionnaires (N = 52)

	Score (+SD)
Pain (VAS)*	5.7 + 2.1
Restrictions in activities in daily living (VAS) *	5.6 + 2.2
Total score questionnaires	29.4 + 13.2
Walking stairs	5.6 + 2.5
Walking ability	11.9 + 5.6
Rising and sitting	11.9 + 6.5

* VAS: Visual Analogue Scale (0 = no pain, no restriction, 10 = worst pain, maximal restriction)

### Concurrent validity for pain

**Figure 2:**
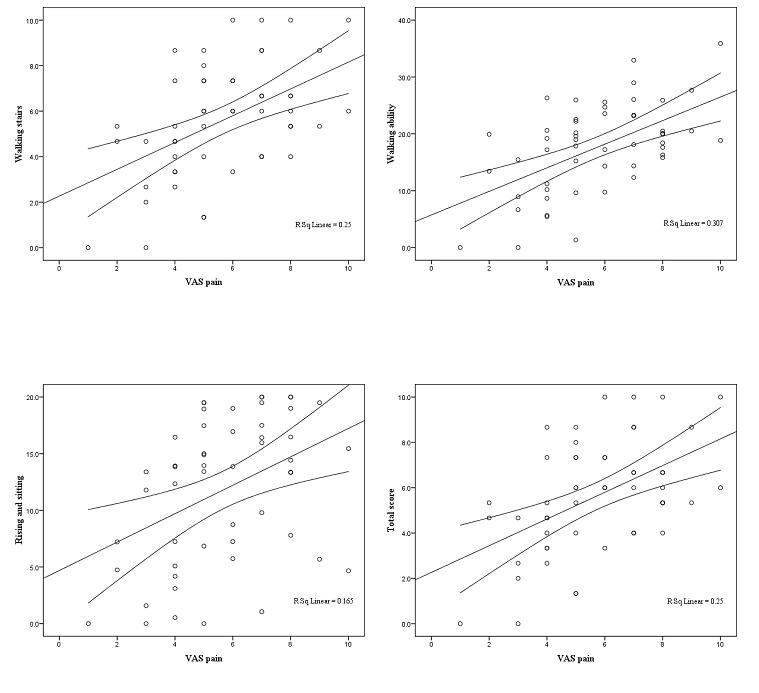
**Linear regression lines with 95% prediction intervals for means (pain)**.

VAS pain scores explained a considerable amount of variability of the total score (R^2 ^= 0.25). Higher VAS pain scores indicated higher total functional limitation (coefficient or slope = 3.33 (95% CI 1.82 to 4.84;p < 0.001)). These results were consistent within the subdomains walking stairs (slope = 0.59 (95% CI 0.30 to 0.88; p < 0.001)), walking ability (slope = 1.49 (95% 0.85 to 2.21; p < 0.001)), and raising and sitting (slope = 1.25 (95% CI 0.45 to 2.06; p = 0.003)). For details see table 3 and figure 2.

**Table 3:** Concurrent validity compared with pain (Visual Analogue Scale) (N = 52)

Domain	Slope *	95%CI	p-Value	**R**^**2**^
Walking stairs	0.59	0.30-0.88	<0.001	0.25
Walking ability	1.49	0.85-2.12	<0.001	0.31
Rising and sitting	1.25	0.45-2.06	0.003	0.17
Total score	3.33	1.82-4.84	<0.001	0.25

* indicating strength of association between Visual Analogue Scale scores and questionnaire domains (from regression analysis)

### Concurrent validity for activities in daily living

VAS ADL scores explained a substantial amount of variability of the total score (R^2 ^= 0.37). Higher VAS ADL scores indicated higher total functional limitation (slope = 8.77 (95% CI 2.32 to 5.04; p < 0.001)). These results were consistent across the subdomains walking stairs (slope = 1.43 (95% CI 0.51 to 0.99; p < 0.001)), walking ability (slope = 2.61 (95% CI 1.10 to 2.23; p = < 0.001)) and raising and sitting (slope = 4.47 (95% CI 0.50 to 2.03; p < 0.02)).

**Figure 3:**
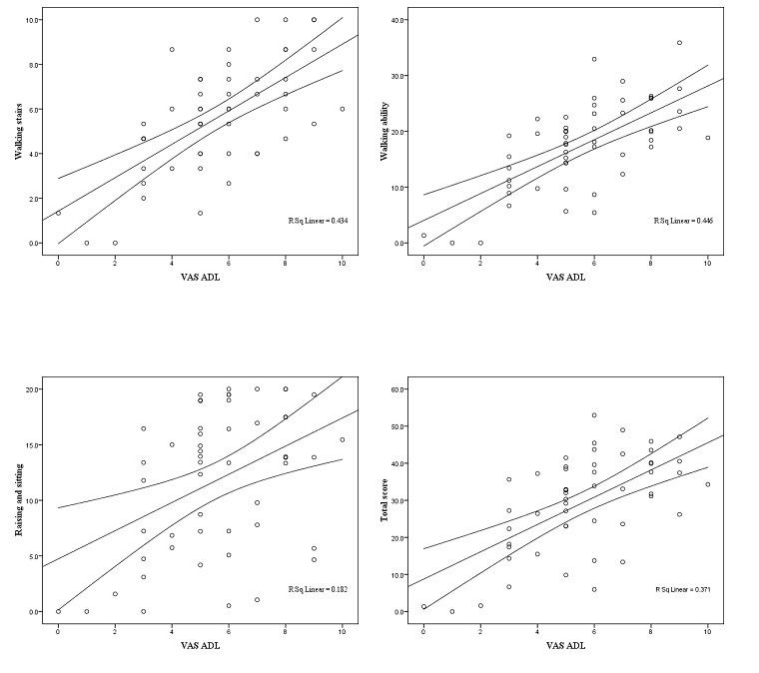
**Linear regression lines with 95% prediction intervals for means (ADL)**.

For details see table 4 and figure 3.

**Table 4:** Concurrent validity compared with ADL (Visual Analogue Scale) (N = 52)

Domain	Slope *	95%CI	p-Value	**R**^**2**^
Walking stairs	1.43	0.51-0.99	<0.001	0.43
Walking ability	2.61	1.10-2.23	<0.001	0.45
Rising and sitting	4.74	0.50-2.03	0.002	0.13
Total score	8.77	2.32-5.04	<0.001	0.37

* indicating strength of association between VAS scores and questionnaire domains (From regression analysis)

## Discussion

### Main findings

We successfully translated the Walking Stairs, Walking Ability and Rising & Sitting Questionnaires into German. Assessing its concurrent validity we found that the German instrument adequately represents activity limitations in daily living and pain in patients with CRPS 1 of the lower extremity. Score values were positively correlated with VAS values for pain and activities in daily living. The correlation of the total score of the three questionnaires was better with VAS ADL than VAS pain. We hypothesize that this difference can be explained by the fact, that pain is a different construct than activity [16].

The translation process itself had no issues of concern, all forward and backward translations were consistent with each other and with the original version. We followed the rigorous translation method proposed by Wild et al. [15], which consisted of a forward and backward translation by professional translators, and by a consensus meeting between researchers. By applying this robust methodology we ensured that the content, integrity and essence of the questionnaires items are maintained and expressed clearly and accurately from one language to another.

### Strength and limitations

To our knowledge, this is the first German translation and external validation of the original Dutch version of the Walking Stairs, Walking Ability and Rising & Sitting Questionnaires allowing the standardized measurement of activity limitations of patients suffering from CRPS 1 of the lower extremity. Another strength is the methods we applied to derive the translated version of the three questionnaires. Our study also has some limitations. First, since diagnosis of CRPS 1 is still a matter of debate our sample might not be representative for a larger CRPS 1 population. The diagnosis of CRPS 1 is based on clinical findings (including sensory, autonomic, motor and trophic changes) and the fulfilment of established diagnostic criteria [17]. We only included patients fulfilling the criteria established by the International Association for the Study of Pain (IASP) [18] in all participants. However, these IASP criteria have been criticized because they are symptom based and show a low specificity [19]. Second, unlike the upper extremities, an instrument for lower extremities was not available to assess the criterion validity of the three questionnaires. Therefore, we had to validate them by assessing the concurrent validity in respect of self reported activity limitations in daily living and pain. However, the correlation between pain and activity level is known to be low in chronic musculoskeletal disability (e.g. [20]). In addition, we are not aware that the reliabil-ity and validity for measuring activity limitations with the VAS have been studied. These considerations also limit the quality of this study. Third, a further limitation might be the fact that we did not look at content validity by coding to the International Classification of Functioning, Disability and Health (ICF), expert content or other CRPS 1 constructs.

### Implications for practice

This validated German version will help to determine the disability of patients suffering from CRPS 1 of the lower extremity in German speaking countries in clinical practice as well as in research. This is important, if these questionnaires will be used to document follow up in longitudinal studies or intervention studies [21]. In addition, it allows a comparison of the results of studies from different origins. In particular, the German versions of the German Walking Stairs, Walking Ability and Rising and Sitting Questionnaires allow us now to collect data for the Swiss CRPS 1 cohort study [21] and to compare the results with our Dutch collaborators within the TREND consortium (Trauma RElated Neuronal Dysfunction, http://www.trendconsortium.nl).

Investigators in German-speaking countries now have the possibility to assess physical activity and limitations in daily living in patients with CRPS 1 of the lower extremity.

Information about patients' disability can be used to enhance clinical decision making and to observe the course of the condition.

## Conclusions

Our study demonstrates a sufficient concurrent validity for the German versions of the Walking Stairs, Walking Ability and Rising and Sitting Questionnaires for the use in clinical practice as well as research. We would like to propagate their use in clinical practice and research alike.

## Competing interests

The authors declare that they have no competing interests.

## Authors' contributions

All authors participated in the study design. FB and LMB drafted the protocol and the manuscript. CH and AL assisted in patient recruiting. RK and FB obtained funding. CH, AGHK, RSGMP and JM critically reviewed the protocol and the manuscript.

## Pre-publication history

The pre-publication history for this paper can be accessed here:

http://www.biomedcentral.com/1471-2474/11/108/prepub
